# Biochemical Properties of Two *Plasmodium malariae* Cysteine Proteases, Malapain-2 and Malapain-4

**DOI:** 10.3390/microorganisms10010193

**Published:** 2022-01-16

**Authors:** Hương Giang Lê, Jung-Mi Kang, Tuấn Cường Võ, Won Gi Yoo, Kon Ho Lee, Byoung-Kuk Na

**Affiliations:** 1 Department of Parasitology and Tropical Medicine, Institute of Health Sciences, Gyeongsang National University College of Medicine, Jinju 52727, Korea; gianglee291994@gmail.com (H.G.L.); gjm9951001@hanmail.net (J.-M.K.); vtcuong241@gmail.com (T.C.V.); wgyoo@gnu.ac.kr (W.G.Y.); 2 Department of Convergence Medical Science, Gyeongsang National University, Jinju 52727, Korea; lkh@gnu.ac.kr; 3 Department of Microbiology, Gyeongsang National University College of Medicine, Jinju 52727, Korea

**Keywords:** *Plasmodium malariae*, cysteine protease, hemoglobin, erythrocyte skeletal proteins, malapain

## Abstract

Cysteine proteases belonging to the falcipain (FP) family play a pivotal role in the biology of malaria parasites and have been extensively investigated as potential antimalarial drug targets. Three paralogous FP-family cysteine proteases of *Plasmodium malariae*, termed malapains 2–4 (MP2–4), were identified in PlasmoDB. The three MPs share similar structural properties with the FP-2/FP-3 subfamily enzymes and exhibit a close phylogenetic lineage with vivapains (VXs) and knowpains (KPs), FP orthologues of *P. vivax* and *P. knowlesi*. Recombinant MP-2 and MP-4 were produced in a bacterial expression system, and their biochemical properties were characterized. Both recombinant MP-2 and MP-4 showed enzyme activity across a broad range of pH values with an optimum activity at pH 5.0 and relative stability at neutral pHs. Similar to the FP-2/FP-3 subfamily enzymes in other *Plasmodium* species, recombinant MP-2 and MP-4 effectively hydrolyzed hemoglobin at acidic pHs. They also degraded erythrocyte cytoskeletal proteins, such as spectrin and band 3, at a neutral pH. These results imply that MP-2 and MP-4 are redundant hemoglobinases of *P. malariae* and may also participate in merozoite egression by degrading erythrocyte cytoskeletal proteins. However, compared with other FP-2/FP-3 enzymes, MP-2 showed a strong preference for arginine at the P2 position. Meanwhile, MP-4 showed a primary preference for leucine at the P2 position but a partial preference for phenylalanine. These different substrate preferences of MPs underscore careful consideration in the design of optimized inhibitors targeting the FP-family cysteine proteases of human malaria parasites.

## 1. Introduction

Falcipains (FPs), a family of papain-like cysteine proteases of *Plasmodium falciparum*, have been extensively investigated due to their critical roles in parasite biology. FPs are principal cysteine proteases mainly expressed in the blood stage of *P. falciparum* and are involved in hemoglobin hydrolysis [1,2,3,4], erythrocyte rupture [5], and erythrocyte invasion by the parasites [6]. FP-1 is also proposed to play a role in oocyst production during gametogenesis [6]. Due to their crucial biological functions, FPs represent attractive targets for antimalarial drugs [7,8]. Cysteine protease inhibitors block hemoglobin degradation in the parasites, resulting in the accumulation of undigested hemoglobin in the food vacuole and eventually impeding parasite development [9,10]. Therefore, the design of specific inhibitors for FPs as potential drug candidates involved extensive efforts [11,12,13].

Five species of *Plasmodium* are known to infect humans and cause malaria. Although their biological properties and clinical features differ, frequent mixed infections of *Plasmodium* species in humans require drugs with a broad antimalarial spectrum against all species of human malaria parasites [14,15]. Therefore, drug design strategies targeting FPs should ideally be extended to orthologous enzymes of other human-infecting *Plasmodium* species to develop broadly effective inhibitors for the FP-family enzymes. In this respect, it is important to understand the biochemical features of the FP-family enzymes in all human-infecting *Plasmodium* species. Orthologous enzymes of the FPs have been identified in other human-infecting *Plasmodium* species, such as *P. vivax* (vivapains, VXs) and *P. knowlesi* (knowpains, KPs), and their biochemical and biological properties have been partially determined [16,17,18]. These FP-family cysteine proteases share highly similar biochemical and structural properties with FPs; however, few unusual features in substrate specificity were also identified [16,17,18].

*Plasmodium malariae* is one of the human malaria parasites contributing to the morbidity of malaria worldwide. Although the parasite species shows narrower prevalence and milder clinical features than *P. falciparum* and *P. vivax*, it can cause chronic low-grade infections that may persist for decades, associated with anemia and nephrotic syndromes [19,20]. Chloroquine-resistant *P. malariae* has also been reported in Indonesia [21]. However, the nature of the FP-family enzymes in the parasite is unknown. In this study, we characterized the biochemical properties of two cysteine proteases belonging to the FP-2/FP-3 subfamily in *P. malariae*: malapain-2 (MP-2) and -4 (MP-4). Although the two MPs share similar structural and biochemical features with other FP-2/FP-3 subfamily enzymes, they also differ in substrate preference. The results of this study provide an important advance to our current perception of drug targets in human malaria parasites.

## 2. Materials and Methods

### 2.1. Identification and Cloning of Genes Encoding Malapains (MPs)

The genes encoding three MPs (MP-2, MP-3, and MP-4) were identified by data mining of PlasmoDB (https://plasmodb.org; accessed on 9 May 2019): MP-2 (Gene ID: PmUG01_09024600), MP-3 (Gene ID: PmUG01_09024700), and MP-4 (Gene ID: PmUG01_ 09024800). Specific primers flanking the open reading frame (ORF) of each MP gene were designed. Each MP gene was amplified via polymerase chain reaction (PCR) of the *P. malariae* genomic DNA. The nucleotide sequences of the primers were as follows: MP-2 (5′-ATGGAGTATCACGTGGAGTATTCTAAT-3′ and 5′-CTACTCAACTAAAGGAATATA- CGCTTC-3′), MP-3 (5′-ATGGAGTACCACATACACTACTCATCA-3′ and 5′-CTAAATA-TCATCAATAATAGCGGTGAA-3′), and MP-4 (5′-ATGGAGTATTCTAAGGATAAAT- ATGTC-3′ and 5′-CTAATCAATCATAGCAATTATCGCTTC-3′). Each PCR product was purified and ligated into the T&A cloning vector (Real Biotech Corporation, Banqiao City, Taiwan), respectively. The *Escherichia coli* DH5α competent cells were transformed with the ligation mixture separately, and the positive clones were selected by colony PCR using the primers indicated above. The nucleotide sequence of each MP was confirmed by automatic DNA sequencing. The primary structure of the deduced amino acid sequences of MPs was analyzed with a DNASTAR package (DNASTAR, Madison, WI, USA) and GENEDOC software (https://genedoc.software.informer.com; accessed on 7 August 2020). The phylogenetic tree was generated based on the sequences of mature domains of FP-family enzymes, papain (Genbank accession number: M15203.1), and human cathepsin L (GenBank accession number: BC012612.1), with MEGA6 (http://www.megasoftware.net; accessed on 2 September 2020), using a neighbor-joining algorithm with 1,000 bootstrap replications.

### 2.2. Expression, Purification, and Refolding of Recombinant MPs

The DNA fragment flanking the mature region and a portion of pro-domain of each MP was amplified by PCR with specific primers as follows: MP-2 (5′-GGATCCGAGAT- GCAAGAGAAATTTCTTATT-3′, contains 5′ *Bam*HI site and 5′-AAGCTTCTACTCAA- CTAAAGGAATATACGC-3′, contains 5′ *Hind*III site); MP-3 (5′-GGATCCGAAATGCAA- CAAAAGTTTCTT-3′, contains 5′ *Bam*HI site and 5′- AAGCTTCTAAATATCATCAATA-ATAGC-3′, contains 5′ *Hind*III site); and MP-4 (5′-GGATCCGTTATGCAAGAAAAG- TTCCTTATC-3′, contains 5′ *Bam*HI site and 5′-AAGCTTCTAATCAATCATAGCAAT- TATCGC-3′, contains 5′ *Hind*III site). Each PCR product was subcloned into the T&A cloning vector (Real Biotech Corporation) and transformed into *E. coli* DH5α. Each resulting plasmid DNA was digested with appropriate restriction enzymes, ligated into the pQE-9 expression vector (Qiagen, Hilden, Germany), and transformed into *E. coli* M15 (pREP4) cells (Qiagen). The expression of recombinant MP was induced with isopropyl-1-thio-β-_D_-galactopyranoside (IPTG; 1 mM of final concentration) for 4 h at 37 °C with gentle shaking at 250 rpm for aeration. The bacteria were harvested by centrifugation at 7000 rpm for 10 min at room temperature and suspended in 8 M urea lysis buffer (8 M urea, 100 mM NaH_2_PO_4_, 10 mM Tris, 20 mM imidazole, pH 8.0) overnight. Each recombinant MP was purified via nickel–nitrilotriacetic acid (Ni–NTA) chromatography (Qiagen), following the manufacturer’s instructions. The purification and purity of recombinant proteins were determined via sodium dodecyl sulfate–polyacrylamide gel electrophoresis (SDS–PAGE). Optimal refolding conditions of each recombinant MP were assessed by refolding the purified proteins in different refolding conditions [22]. For large-scale refolding, the purified recombinant protein (200 mg) was slowly added to the optimized refolding buffer (100 mM Tris-HCl, pH 8.0 supplemented with 1 mM EDTA, 250 mM _L_-arginine, 30% glycerol, 2 mM reduced glutathione (GSH), and 1 mM oxidized glutathione (GSSG)) and incubated at 4 °C overnight with gentle stirring. The refolded protein was concentrated using the Centriprep (10 kDa cut-off; Merck Millipore, Burlington, MA, USA). To induce autocatalytic processing of the MP into an active mature enzyme, the pH was adjusted to 5.0 in the presence of 10 mM dithiothreitol (DTT) and incubated at 37 °C for 2 h. Aliquots were obtained every 15 min for enzyme assay and SDS–PAGE.

### 2.3. Enzyme Activity Assay

Enzyme activity was assayed via hydrolysis of fluorogenic peptide substrates, including benzyloxycarbonyl-_L_-leucyl-_L_-arginine 4-methyl-coumaryl-7-amide (Z-LR-MCA), Z-_L_-phenylalanyl-_L_-arginine MCA (Z-FR-MCA), and Z-_L_-arginyl-_L_-arginine MCA (Z-RR-MCA) (Peptide International, Louisville, KY, USA). The enzyme (20 nM) was added to the assay buffer (10 nM of each peptide substrate, 10 mM DTT, 100 mM sodium acetate (pH 5.0)), and the release of fluorescence was detected at excitation and emission wavelengths of 355 nm and 460 nm, respectively, over 20 min at 37 °C using Fluoroskan Ascent FL (Thermo Fisher Scientific, Vantaa, Finland).

### 2.4. Biochemical Properties and Kinetics of MP-2 and MP-4

The optimal pH for the maximum activity of MP-2 and MP-4 was analyzed. Each enzyme was added to different pH buffers (100 mM sodium acetate (pH 4.0–5.5), sodium phosphate (pH 6.0–6.5) or Tris-HCl (7.0–7.5)) containing 10 mM DTT and 10 nM of each fluorogenic peptide substrate, and the hydrolysis of the substrate was measured as described above. The pH stability of MP-2 and MP-4 was ascertained by incubating each enzyme in the pH buffers (pH 4.0–7.0) at 37 °C for indicated time points, and the residual enzyme activity was assayed. The effect of reducing agents on enzyme activity was determined using a 100 mM sodium acetate buffer (pH 5.0) containing 10 nM of appropriate substrates and various concentrations of DTT or reduced glutathione (GSH). Active site titration assay was performed using trans-epoxysuccinyl-_L_-leucylamido(4-guanidino)butane (E-64) described previously [23]. Kinetic analysis was performed by incubating a constant concentration of enzymes (10 nM) with varying concentrations (0 to 600 nM) of each peptide substrate, including Z-LR-MCA, Z-FR-MCA, or Z-RR-MCA, in 100 mM sodium acetate (pH 5.0) containing 10 mM DTT. The released fluorescence was monitored at 37 °C for 20 min.

### 2.5. N-Terminal Amino Acid Sequencing

The fully processed recombinant MP-2 and MP-4 proteins were separated by 12% SDS–PAGE and transferred to a polyvinylidene difluoride (PVDF) membrane (Merck Millipore). The membrane was stained with Coomassie blue and destained. The protein band was excised, followed by N-terminal amino acid sequencing using an ABI model 477A protein sequencer (Applied Biosystems, Waltham, MA, USA) and an ABI model 120A PTH analyzer (Applied Biosystems) at the Korea Basic Science Institute (Daejeon, Korea).

### 2.6. Hydrolysis of Human Hemoglobin

The hydrolysis of hemoglobin by MP-2 and MP-4 was assessed by incubating 50 nM of each enzyme with human hemoglobin (10 µg: Sigma, St. Louis, MO, USA) in different pH buffers ranging from 4.0 to 7.0 at 37 °C for 4 h in the presence of 1 mM DTT. Time-dependent hemoglobin hydrolysis by the two enzymes was also analyzed by incubating each enzyme (50 nM) with hemoglobin (10 µg) in 100 mM sodium acetate (pH 5.0). To evaluate the effect of reducing agents (GSH and DTT) on the hydrolysis of hemoglobin, each enzyme was mixed with hemoglobin (10 µg) in 100 mM sodium acetate (pH 5.0) with varying concentrations of GSH or DTT and incubated at 37 °C for 4 h. The reactions were terminated by adding the SDS–PAGE sample buffer at indicated time intervals and analyzed via SDS–PAGE.

### 2.7. Hydrolysis of Erythrocyte Cytoskeletal Proteins

Erythrocyte ghosts were isolated from fresh human blood via hypotonic lysis of erythrocytes in 20 mM sodium phosphate (pH 7.4) and supplemented with 0.1% saponin, as described previously [24]. The purified ghosts were incubated with 50 nM of each enzyme at pH 5.0 or 7.0 at 37 °C for 4 h. The mixtures were analyzed by SDS–PAGE, followed by Coomassie blue staining. For immunoblot analysis, the mixtures were transferred to nitrocellulose membranes (0.45 µm: Bio-Rad, Hercules, CA, USA), and the membranes were blocked with phosphate-buffered saline (PBS, pH 7.4), supplemented with 0.05% Tween 20 and 5% (*w*/*v*) skim milk for 1 h at room temperature. The membranes were rinsed with PBS, supplemented with 0.05% Tween 20 (PBST) several times, and incubated with one of the monoclonal antibodies against anti-human spectrin (Sigma, 1:500 dilution), anti-human actin (Sigma, 1:1000 dilution), and anti-human band 3 (Sigma, 1: 5000 dilution) for 3 h at room temperature, respectively. Each membrane was repeatedly washed with PBST and incubated with horseradish peroxidase-conjugated host-specific antibodies (Sigma) for 2 h at room temperature. The immunoreactive bands were visualized with the SuperSignal West Pico PLUS chemiluminescent substrate (Thermo Fisher Scientific).

### 2.8. Statistical Analysis

All enzyme assay data were expressed as the mean values of percent activity ± standard deviation (mean ± SD) from three independent experiments. The data were analyzed using Student’s *t*-test. Differences in mean values were considered statistically significant at *p* < 0.05.

## 3. Results

### 3.1. MPs Showed Close Relationship with VXs and KPs

**Figure 1:**
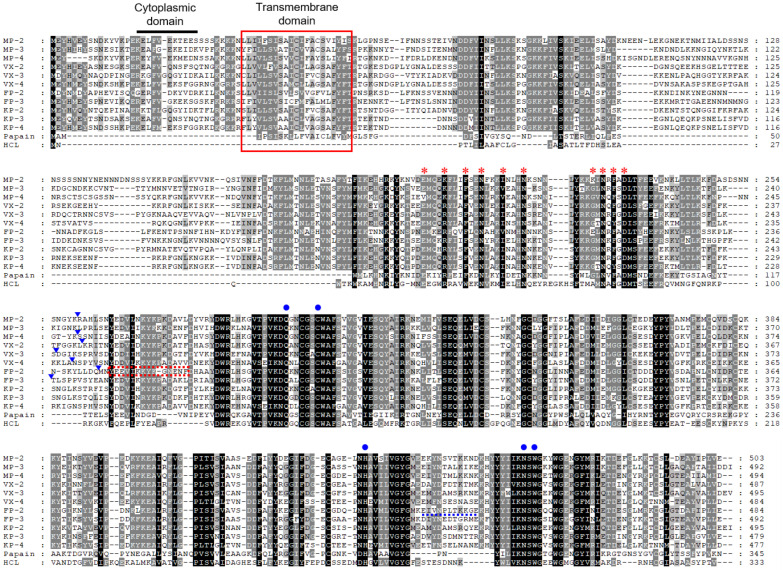
Sequence alignment of MPs and FP-2/FP-3 subfamily enzymes. The deduced amino acid sequences of MPs, VXs, FPs, KPs, papain, and human cathepsin L (HCL) were aligned. Dashes represent gaps introduced to maximize alignment. The predicted cytoplasmic domain is represented as a bold black bar on the sequences. The predicted transmembrane domain is boxed in red. Amino acid residues corresponding to ERFNIN and GNFD motifs, which are conserved pro-domain motifs of papain-like family enzymes, are marked with red asterisks. Blue circles indicate conserved residues for the active site formation. The predicted refolding motif of FP-2 is shown as a red dotted box. The hemoglobin-binding motif is underlined as a blue dotted line. The positions of experimentally confirmed or predicted mature domain processing sites are indicated by blue arrows. Percentage of identity among the sequences is represented as shading: black (>88%), dark grey (75–88%), light grey (37–75%), and no shading (<37%).

**Figure 2:**
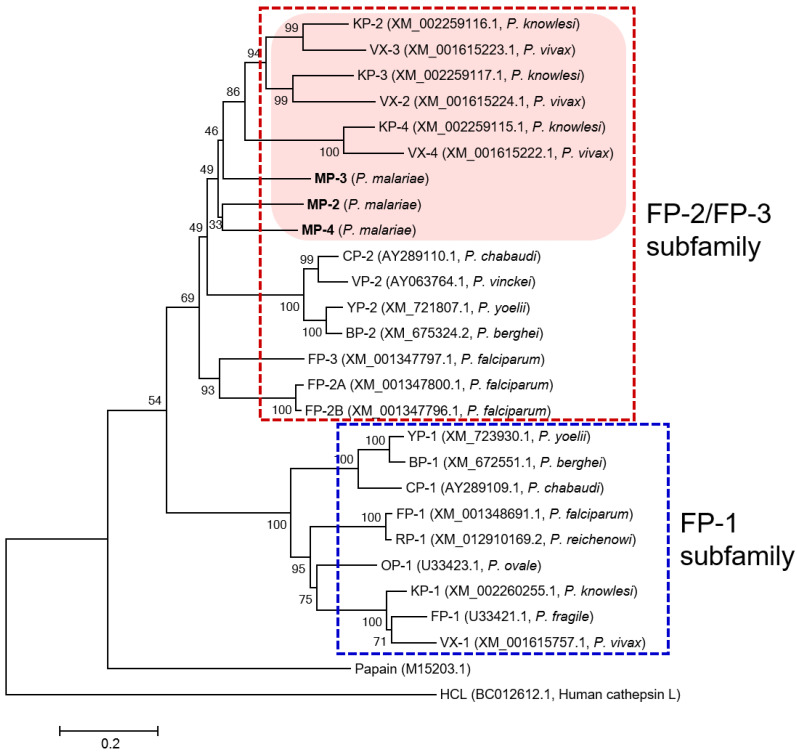
Phylogenetic analysis. The phylogenetic tree was constructed based on the sequences of the mature domain of each enzyme by MEGA6 using neighbor-joining algorithm with 1000 bootstrap replications. FP-family enzymes were clustered into two distinct clades: FP-2/FP-3 subfamily and FP-1 subfamily. MPs showed a closer lineage with VXs and KPs distinct from FPs.

Three paralogous sequences for FP-family cysteine proteases of *P. malariae*, MP-2, MP-3, and MP-4, were identified in the *P. malariae* sequences in the PlasmoDB database. The three MPs exhibited unique structural features of the FP-family enzymes, including a cytoplasmic domain and a transmembrane domain in the signal motif, the refolding domain motif, and the hemoglobin-binding arm at the C-terminal region between conserved catalytic residues (Figure 1). The amino acid residues (Q, C, H, N, and W) constituting the active site and the inhibitory motifs ERFNIN and GNFD were also well conserved in the sequences. Analysis of amino acid sequence alignment of MPs revealed a similar degree of sequence identity with each other: MP-2 and MP-3 (51.7%), MP-2 and MP-4 (60.5%), and MP-3 and MP-4 (52.7%). They showed a high degree of sequence identity with the FP-2/FP-3 subfamily enzymes in other *Plasmodium* species, including VXs (47.6–57.3%), FPs (47.2–54.7%), and KPs (48.0–59.8%) (Table 1). The phylogenetic analysis of MPs and FP-family enzymes revealed they clustered into two distinct clades: the FP-2/FP-3 subfamily and the FP-1 subfamily (Figure 2). The three MPs were clustered into the FP-2/FP-3 subfamily and formed clades with VXs and KPs distinct from FPs within the FP-2/FP-3 subfamily. Within each subfamily, the FP-family enzymes derived from rodent malaria parasites formed distinct clades compared with those of human malaria parasites.

### 3.2. Recombinant MP-2 and MP-4 Were Successfully Produced by E. coli

**Figure 3:**
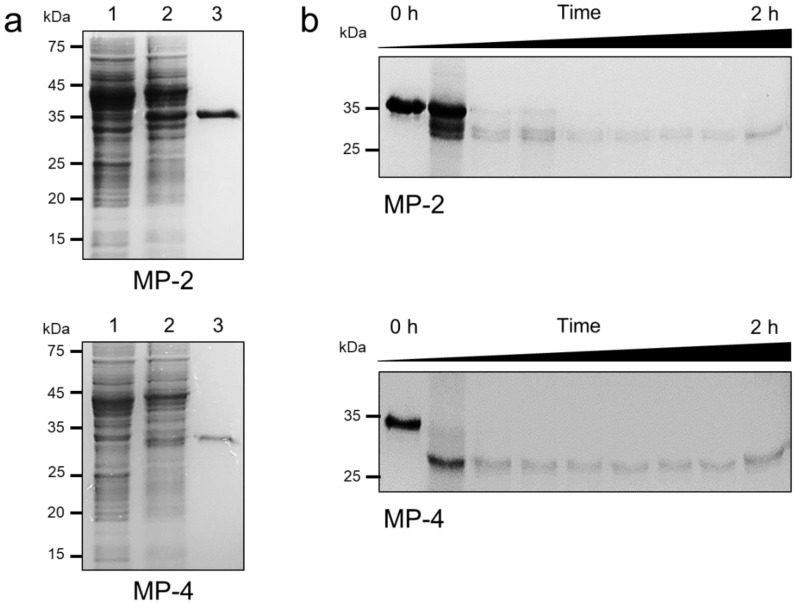
Expression, purification, and autocatalytic processing of MP-2 and MP-4. (**a**) Recombinant MP-2 and MP-4 proteins were expressed in *E. coli*, purified via Ni–NTA affinity chromatography, analyzed by 12% SDS–PAGE, and stained with Coomassie blue. Lane 1, non-induced *E. coli* lysate; lane 2, IPTG-induced *E. coli* lysate; lane 3, recombinant protein purified via Ni–NTA affinity chromatography. (**b**) Autocatalytic processing of refolded MP-2 and MP-4 proteins. Each refolded protein was activated in acidic conditions, and the aliquots taken at every 15 min were analyzed by 12% SDS–PAGE.

Constructs flanking the C-terminal portion of the pro-domain and mature domain of each MP were generated and expressed in *E. coli*. Recombinant MP-2 and MP-4 were successfully expressed in *E. coli* as insoluble proteins with expected molecular masses of about 36 kDa for MP-2 and 34 kDa for MP-4 (Figure 3). Meanwhile, in the case of MP-3, the recombinant protein was not successfully expressed in *E. coli*, despite repeated trials under different expression conditions. Therefore, MP-3 was excluded from further analysis. Recombinant MP-2 and MP-4 were purified from solubilized inclusion bodies via Ni–NTA affinity chromatography (Figure 3a). The refolded recombinant MP-2 and MP-4 proteins underwent autocatalytic processing to yield mature enzymes upon exposure to acidic conditions in the presence of a reducing agent (Figure 3b). The mature MP-2 and MP-4 were approximately 28 kDa and 27 kDa, respectively. The N-terminal amino acid sequences of the fully processed mature MP-2 and MP-4 proteins were determined as RAHLS and SSNIISY, respectively, via N-terminal amino acid sequencing analysis (Figure 1).

### 3.3. MP-2 and MP-4 Showed Different Preference for Peptide Substrates

**Figure 4:**
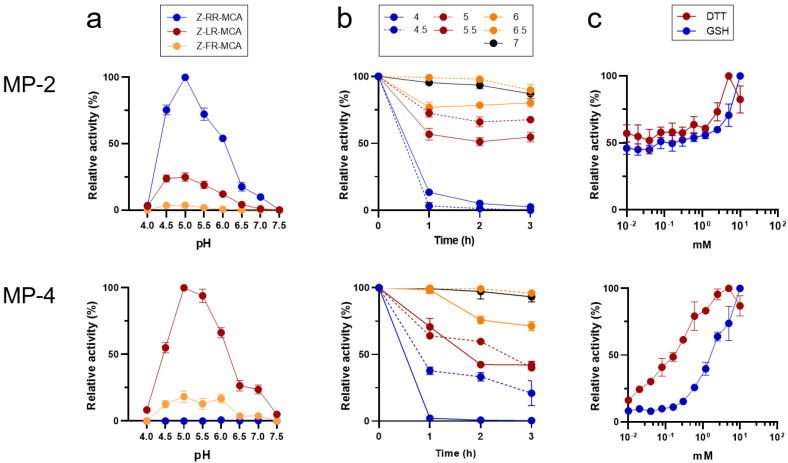
Biochemical properties of recombinant MP-2 and MP-4. (**a**) Hydrolysis of dipeptidyl substrates. Activity of each enzyme against each substrate was assayed in 100 mM sodium acetate (pH 4.0–5.5), sodium phosphate (6.0–6.5), or Tris-HCl (pH 7.0–7.5) supplemented with 10 mM DTT. Maximal activity was presented as 100%. (**b**) Enzyme stability. Each enzyme was incubated in different pH buffers for the indicated time, and the residual enzyme activity was assayed. For MP-2, Z-RR-MCA was used as a substrate. Meanwhile, Z-LR-MCA was used as a substrate for MP-4. (**c**) Effect of reducing agents. Activity of each enzyme was assayed in 100 mM sodium acetate buffer (pH 5.0) in the presence or absence of different concentrations of DTT or GSH. Enzyme activity was assayed with Z-RR-MCA and Z-LR-MCA for MP-2 and MP-4, respectively.

Both MP-2 and MP-4 showed enzyme activities in a broad range of pH values ranging from 4.5 to 6.0, with an optimum activity at pH 5.0 (Figure 4a). However, the two enzymes showed different substrate preferences. MP-2 hydrolyzed all the tested dipeptidyl substrates, Z-RR-MCA, Z-LR-MCA, and Z-FR-MCA (Figure 4a). However, MP-4 hydrolyzed Z-LR-MCA and Z-FR-MCA, but not Z-RR-MCA. Kinetic analyses of the two enzymes against each dipeptidyl substrate also revealed different substrate preferences (Table 2). Both MP-2 and MP-4 were relatively stable at neutral pHs with up to 75% of activity intact even after 3 h of incubation. However, both enzymes were unstable in acidic conditions, resulting in a rapid loss of enzyme activities, particularly at a pH less than 4.5 (Figure 4b). The activities of MP-2 and MP-4 were enhanced by the reducing agents, DTT and GSH, in a dose-dependent manner (Figure 4c).

### 3.4. MP-2 and MP-4 Hydrolyzed Hemoglobin and Erythrocyte Cytoskeletal Proteins

**Figure 5:**
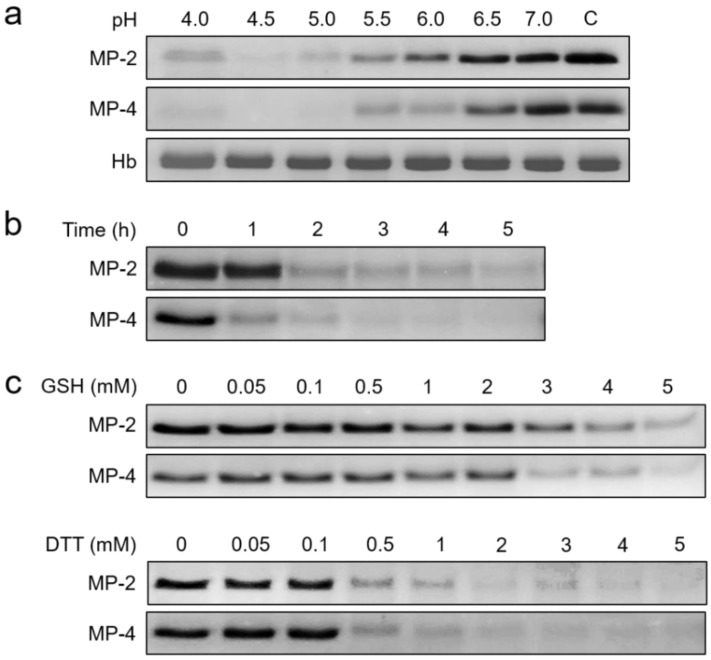
Hemoglobin hydrolysis by MP-2 and MP-4. Human hemoglobin was incubated with each enzyme and was resolved by SDS–PAGE followed by Coomassie blue staining. (**a**) Effect of pH. Each MP (50 nM) was incubated with hemoglobin (10 µg) in different pH buffers (pH 4.0–7.0) containing 1 mM DTT at 37 °C for 4 h, and the mixtures were analyzed by SDS–PAGE. C, hemoglobin control without enzyme. Hb, only hemoglobin. (**b**) Time-dependent hydrolysis. Human hemoglobin (10 µg) was incubated with 50 nM of each enzyme in 100 mM sodium acetate (pH 5.0) supplemented with 1 mM DTT at 37 °C, and aliquots were withdrawn at indicated time points and analyzed via SDS–PAGE. (**c**) Effect of reducing conditions. Each enzyme (50 nM) was incubated with human hemoglobin (10 µg) in 100 mM sodium acetate (pH 5.0) containing different concentrations of GSH (upper panel) or DTT (lower panel) at 37 °C for 4 h and analyzed via SDS–PAGE.

**Figure 6:**
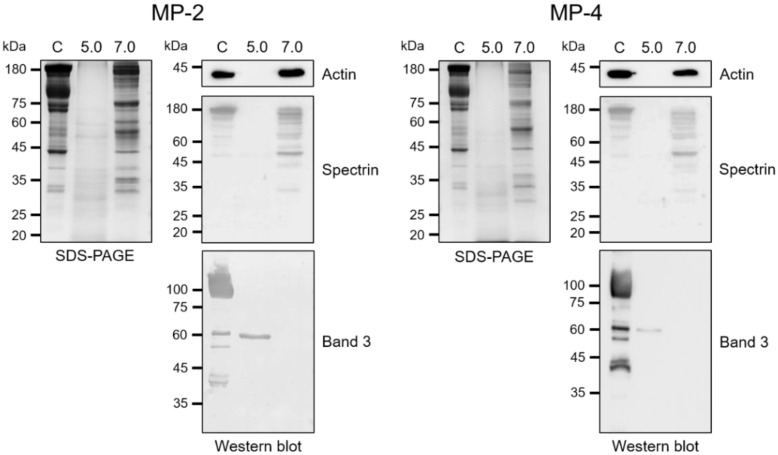
Degradations of erythrocyte cytoskeletal proteins by MP-2 and MP-4. Erythrocyte ghost prepared from fresh human erythrocytes were incubated with MP-2 (50 nM) or MP-4 (50 nM) in 100 mM sodium acetate (pH 5.0) or 100 mM Tris-HCl (pH 7.0) supplemented with 1 mM DTT at 37 °C for 4 h. The mixtures were analyzed via SDS–PAGE or immunoblot. For immunoblot, the proteins were transferred onto nitrocellulose membranes and probed with specific monoclonal antibodies against human actin, spectrin, or band 3, respectively. C, erythrocyte ghost control without enzyme.

MP-2 and MP-4 hydrolyzed human hemoglobin at a broad range of pH values (4.0 to 6.0) with an optimum effect at 4.5 (Figure 5a). MP-4 hydrolyzed hemoglobin more effectively than MP-2 (Figure 5b). The hydrolysis of hemoglobin was enhanced by the reducing agents, GSH and DTT, in a dose-dependent manner (Figure 5c). MP-2 and MP-4 also hydrolyzed erythrocyte cytoskeletal proteins, including actin, spectrin, and band 3 (Figure 6). Both enzymes effectively degraded actin, spectrin, and band 3 at pH 5.0, whereas hydrolyses of spectrin and band 3 were also observed in a neutral environment, pH 7.0 (Figure 6).

## 4. Discussion

This study is the first report of the biochemical properties of MPs, the cysteine proteases belonging to the FP-family in the human malaria parasite *P. malariae*. The three MPs, MP-2, MP-3, and MP-4, shared similar structural features with the FP-2/FP-3 subfamily enzymes and showed high levels of sequence identity with the FP-2/FP-3 subfamily enzymes from other human malaria parasites. In particular, MPs showed higher sequence identities with VXs and KPs than FPs, which distinguish these enzymes as a distinct clade in the phylogenetic analysis.

MP-2 and MP-4 shared similar biochemical properties with other FP-2/FP-3 subfamily enzymes [3,4,16,17,18]. Malaria parasites take up the hemoglobin of erythrocytes, degrade it in the acidic food vacuole, and utilize the amino acids derived from hemoglobin for nutrition [25]. FP-2/FP-3 subfamily enzymes are active hemoglobinases of *Plasmodium* parasites based on their principal localization in the food vacuole, strong hemoglobin hydrolytic activity in the acidic food vacuolar environment, and high levels of expressions in trophozoite stages [3,4,16,17,18]. MP-2 and MP-4 also effectively hydrolyzed dipeptidyl peptide substrates and hemoglobin at acidic pHs similar to other characterized FP-2/FP-3 subfamily enzymes, suggesting that MP-2 and MP-4 are competent food vacuole hemogolobinases of *P. malariae*, even though the localization of the two enzymes in the parasite has yet to be elucidated. FP-2/FP-3 enzymes also participate in merozoite egress by mediating erythrocyte rupture via degradation of erythrocyte cytoskeletal proteins [3,16,17,18]. Similar to other FP-2/FP-3 subfamily enzymes, MP-2 and MP-4 also degraded erythrocyte cytoskeleton proteins, such as spectrin and band 3, at a neural pH, suggesting their potent role in the remodeling of the erythrocyte membrane during the merozoite egression from erythrocytes.

Unlike human cathepsin B and L that prefer Phe or Leu residues at the P2 position in substrates and inhibitors, the FP-2/FP-3 subfamily enzymes show strict or moderate preference for Leu at the position [3,4,16,17,18]. Similar patterns of preference for Leu at the P2 position were also detected in the FP-2/FP-3 subfamily enzymes in rodent malaria parasites: vinckepain-2 for *P. vinckei* and berghepain-2 for *P. berghei* [26,27]. This selectivity of FP-2/FP-3 enzymes for Leu at the P2 position renders them attractive targets for specific inhibitor exploitation, suggesting therapeutic effectiveness. MPs share similar, but not identical, P2 preference with other FP-2/FP-3 subfamily enzymes. Similar to VX-2, VX-3, KP-2, and KP-3 [16,18], MP-4 showed a strong preference for Leu at the P2 position, but it also hydrolyzed Z-FR-AMC unlike VX-2, VX-3, KP-2, and KP-4. Meanwhile, MP-2 hydrolyzed all three dipeptidyl peptide substrates tested at acidic pH similar to KP-4 with the preference of Arg > Leu > Phe. FP-2, FP-3, and VX-4 also hydrolyzed the three dipeptidyl substrates but favored Leu > Phe > Arg [4,17]. These findings suggest that the FP-2/FP-3 subfamily enzymes showed slightly different substrate preferences for the P2 position, although the general biochemical properties were highly similar. The molecular mechanism underlying the different substrate preferences of MPs has yet to be elucidated, and further structural analysis of MPs is underway.

Functional expression and characterization of MPs widen our understanding of the nature of the enzymes. High sequence identities and similar biochemical properties of MP-2 and MP-4 compared to FP-2/FP-3 subfamily enzymes derived from other human malaria parasites suggest that MP-2 and MP-4 are competent hemoglobinases of *P. malariae*. The fact that MP-2 and MP-4 share strict or at least moderate selective preference for Leu at the P2 position with FP-2/FP-3 subfamily enzymes from other human malaria parasites supports the notion that a common inhibitor of these enzymes is an effective therapeutic strategy against all human malaria parasites by inhibiting hemoglobin hydrolysis and impeding survival of the parasites. However, the minute differences in substrate preference of the FP-2/FP-3 subfamily enzymes in human malaria parasites should be carefully analyzed to design optimized inhibitors targeting the enzymes. Given that the in vitro culture of *P. malariae* has yet to be successfully established, recombinant MPs enable convenient screening of the potential inhibitors of the FP-2/FP-3 subfamily enzymes and facilitate high-throughput screening of a broad spectrum of drug candidates against multiple species of human malaria parasites.

## Figures and Tables

**Table 1:** Sequence identity between different cysteine proteases.

	MP-3	MP-4	VX-2	VX-3	VX-4	FP-2	FP-3	KP-2	KP-3	KP-4	Papain	HCL
**MP-2**	51.7	60.5	51.0	48.0	50.3	47.2	49.6	48.8	48.0	50.9	21.0	20.2
**MP-3**	–	52.7	52.0	59.8	47.6	49.2	49.6	59.8	50.2	49.2	21.2	17.9
**MP-4**	–	–	57.3	50.0	50.7	48.4	54.7	48.4	53.7	49.5	22.1	20.2
**VX-2**	–	–	–	53.9	60.2	48.7	53.1	53.5	71.7	57.6	20.7	19.8
**VX-3**	–	–	–	–	48.5	44.9	47.1	77.8	51.5	48.7	21.0	18.7
**VX-4**	–	–	–	–	–	44.4	49.7	47.9	53.7	78.4	20.3	19.6
**FP-2**	–	–	–	–	–	–	54.3	44.3	45.2	44.4	22.8	20.7
**FP-3**	–	–	–	–	–	–	–	47.3	50.3	49.5	20.9	21.2
**KP-2**	–	–	–	–	–	–	–	–	57.4	49.9	21.0	20.0
**KP-3**	–	–	–	–	–	–	–	–	–	66.4	21.0	20.1
**KP-4**	–	–	–	–	–	–	–	–	–	–	20.4	19.9
**Papain**	–	–	–	–	–	–	–	–	–	–	–	21.7

HCL: human cathepsin L.

**Table 2:** Kinetics of MP-2 and MP-4.

Substrate	*K*_cat_/*K*_m_ (s^−1^ M^−1^)	
	MP-2	MP-4
Z-LR-AMC	3.9 × 10^4^	7.4 × 10^4^
Z-FR-AMC	3.2 × 10^4^	3.8 × 10^4^
Z-RR-AMC	9.2 × 10^4^	NH *

* No hydrolysis.

## Data Availability

The data supporting the conclusions of this article are provided within the article. The original data in the present study are available from the corresponding author upon request.
